# Interprofessional care for Indigenous women during pregnancy and childbirth in light of Complexity Theory

**DOI:** 10.1590/1980-220X-REEUSP-2025-0504en

**Published:** 2026-05-11

**Authors:** Julieli Rosso, Luana Pizarro Meneghello, Silvia Naujorks, Janet Mercedes Arévalo-Ipanaqué, Dirce Stein Backes, Juliana Silveira Colomé

**Affiliations:** 1 Universidade Franciscana, Programa de Pós-Graduação em Saúde Materno-Infantil, Santa Maria, RS, Brazil.; 2 Universidad Peruana Cayetano Heredia, Facultad de Enfermería, Lima, Peru.

**Keywords:** Health of Indigenous Peoples, Perinatal Care, Maternal and Child Health, Interprofessional Relations, Philosophy

## Abstract

**Objective:**

To reflect, in light of complexity theory, on the dynamics of interprofessional healthcare for indigenous women during pregnancy and the postpartum period.

**Method:**

A theoretical-reflective study, using Edgar Morin’s complexity framework, through the tetragram to reveal the encounters, tensions, and possibilities present in interprofessional care in intercultural contexts.

**Results:**

Interprofessional care emerges as a crucial strategy for overcoming the fragmentation of the hegemonic biomedical model. The application of the tetragram has demonstrated that: order resides in traditional practices and biomedical protocols; disorder manifests itself in cultural ruptures and conflicts; interaction occurs in experiences of articulation between different types of knowledge; and organization indicates the construction of more inclusive and adaptive care arrangements.

**Conclusion:**

Complexity theory offers a fruitful lens for rethinking and (re)orienting attention to indigenous women’s health, highlighting the imperative of active listening, valuing traditional knowledge, and building culturally safe, contextualized, and transformative interprofessional practices.

## INTRODUCTION

Indigenous health during pregnancy and childbirth is a field that requires a systemic, sensitive, and integrated understanding of cultural, spiritual, social, and biological dimensions^(1,2)^. In this context, it is necessary to promote interprofessional practices that overcome fragmentation and isolation between professional categories. Such approaches require the development of shared and evolving leadership capable of articulating and promoting the cooperation necessary to respond effectively to the complexity of health care^(3,4)^.

Interprofessional care in health and education is a strategic axis for the construction of training and practical arrangements that articulate different types of knowledge in processes of experimentation, cooperation, and collective production. Interprofessional teams are fundamental in improving the capacity of health systems to meet the health needs of the population^(5,6)^.

Studies show that indigenous women in Brazil have higher rates of maternal and infant morbidity and mortality when compared to the general population, influenced by social determinants such as poverty, social exclusion, educational and racial disparities, geographical barriers, and institutional discrimination^(7,8,9)^. Given the multifaceted nature of these inequalities, a theoretical framework capable of integrating multiple dimensions is necessary.

Considering the interrelationship between cultural, social, institutional, and spiritual factors, it is evident that linear approaches do not account for the complexity involved in the health care of indigenous women. Furthermore, despite advances in public policies, there is a scarcity of studies that address interprofessional health care for indigenous women from the perspective of complexity.

The theory of complexity, proposed by Edgar Morin, offers a robust approach for critically reflecting on interprofessional healthcare for indigenous women, overcoming the fragmented and linear views that predominate in traditional biomedical models. Complexity is a fabric of inseparably associated heterogeneous constituents, which challenges the dichotomy between the one and the multiple and proposes a vision that integrates diversity and interconnection^(10)^. Indigenous health care requires the recognition of different conceptions of life, health, and disease. This demands an ethical stance that overcomes the limits of the universalist biomedical model, which often excludes ancestral knowledge^(11,12,13)^.

It is argued that the complexity framework can support a sensitive, critical, and integrative analysis of interprofessional health care for indigenous women in an intercultural context. Thus, the question arises: how can complexity theory contribute to reflection and the practice of interprofessional health care for indigenous women in the pregnancy-puerperal cycle?

Thus, this theoretical-reflective study, in dialogue with Edgar Morin, seeks to reflect, in light of complexity theory, on the dynamics of interprofessional care for the health of indigenous women in the pregnancy-puerperal cycle.

## METHOD

This is a theoretical-reflective study with a qualitative approach, based on Edgar Morin’s Complex Thought, especially through Morin’s tetragram (order, disorder, interaction, and organization) as an analytical framework to reveal the encounters, tensions, and possibilities present in interprofessional care in intercultural contexts, in the context of the course “Construction of Systemic Thought,” taken as part of doctoral training. Initially conceived as a book chapter, the text was reworked and adapted to the format of a reflective scientific article.

The reflective process was based on the articulation between the central theoretical framework and a corpus of national and international scientific productions related to interprofessional care and indigenous health. These works were selected intentionally and analytically, considering their thematic focus, conceptual relevance, and potential for dialogue with the assumptions of complexity/complex thinking/theory.

Bibliographic searches were conducted in the Virtual Health Library (VHL), covering articles, technical documents, and theoretical works relevant to the field of public health, nursing, and indigenous health. This was not a systematic review, but rather a theoretical survey guided by reflective questioning, seeking to support the argumentative construction and critical analysis proposed.

The study was conducted from August to October 2025, considering publications available up to that time, which contributes to the contextualization and timeliness of the reflection developed. The analytical process consisted of in-depth reading, critical interpretation, and conceptual articulation of the selected works, in continuous dialogue with Morin’s framework, seeking to understand the complexity of health care in contexts marked by interprofessionality and sociocultural diversity.

Thus, this study is based on the theoretical-reflective framework of Edgar Morin’s theory of complexity, in order to provide a foundation for interprofessional healthcare for indigenous women in the pregnancy-puerperal cycle.

## The Complex Thought of Edgar Morin

The theoretical framework adopted is based on the theory of complexity proposed by Edgar Morin, a French philosopher, sociologist, and epistemologist born in 1921. His biography, marked by early losses such as the death of his mother at the age of nine, contributed to a particular sensitivity to the human condition and its multiple dimensions. Throughout his intellectual career, Morin has shown an interest in literature, music, and cinema, moving through different fields of knowledge and consolidating an integrative vision, especially in the human sciences^(14)^.

Complexity is a set of inseparably associated elements, expressed as a fabric of events, actions, interactions, feedback, determinations, and chance occurrences that constitute reality^(15)^. This intertwining involves uncertainties, indeterminacies, and randomness that challenge reductionist or linear views^(16)^.

Complexity theory seeks to integrate aspects often seen as contradictory, such as unity and diversity, continuity and rupture, construction and deconstruction, recognizing that it is not possible to analyze the parts without the whole, nor the whole without the parts. In this sense, complexity should be understood as a problem word, not a solution word: it does not provide ready answers, but invites permanent reflection, critical analysis, and openness to the unexpected^(15)^.

In light of complexity theory, the coexistence of order and disorder is reflected, illustrating this idea with the observation of the starry sky. What initially appears to be a random grouping of stars reveals, upon closer inspection, a cosmic order; at the same time, when considering the expanding universe, one perceives the creative disorder of stars that are born, explode, and die. Thus, order and disorder constitute inseparable dimensions of reality^(15)^.

This concept is fundamental to thinking about the challenges of indigenous health care in the pregnancy-puerperal cycle, marked by a complex web of cultural, spiritual, social, and biological elements. Care for indigenous women cannot be reduced to linear biomedical logic based on technical protocols. It is necessary to recognize the coexistence of multiple orders and disorders, traditional and scientific knowledge, institutional routines and ancestral practices, rights that have been won, and historical violence that is still present^(1,17)^.

The theory of complexity is based on principles that are articulated in the tetralogical circuit or tetragram - order, disorder, interaction, and organization, guiding reflection toward the reconnection of knowledge^(15)^. The principles of complexity theory, proposed by Morin, provide essential foundations for understanding phenomena in their entirety. The systemic principle shows that parts and whole are inseparable, while the holographic principle reveals that each part contains the whole and vice versa, overcoming reductionism and holism. The retroactive and recursive circuits highlight self-regulation and circularity between causes and effects. Autonomy is always dependent on the environment, while the dialogical principle integrates opposing notions that coexist in reality. Finally, the reintroduction of knowledge proposes a reform of thought, recognizing limits, uncertainties, and the need for constant contextualization.

In the field of indigenous health, the principles of Morin’s complexity theory help to understand care in a broader way. The dialogical principle highlights the need to integrate technical knowledge and traditional knowledge, allowing science and ancestry to coexist in dialogue. The recursive principle shows that care practices are not self-contained, but feed back into culture, health, and territory, continuously strengthening community life. The holographic principle reveals that each part, whether the professional, the community, the woman, or the family, contains and reflects the whole, showing that care is simultaneously individual and collective, singular and communal^(16,18)^.

### Morin’s Tetragram and the Indigenous Gravid-Puerperal Cycle

**Figure 1:**
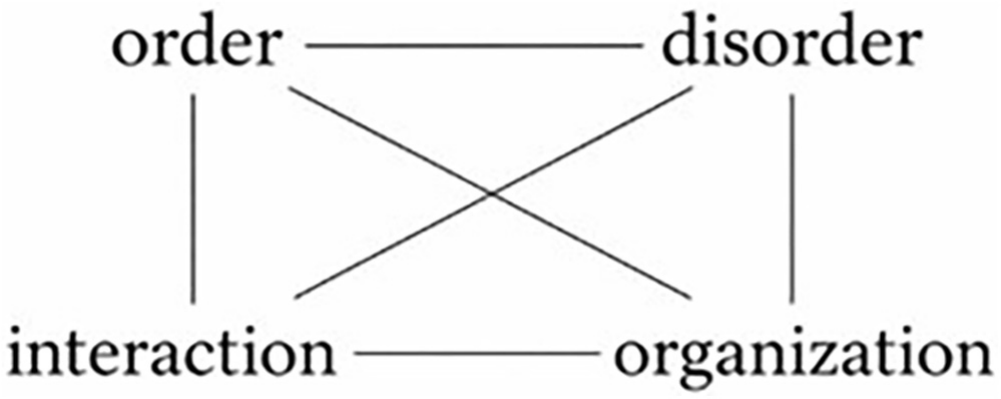
Morin’s tetragram.

Source: Morin^(15:204)^.

In light of complexity theory, the dynamic processes of reality can be understood through the tetragram, a proposition that considers that any living or social system is the result of the interaction between order, disorder, interaction, and organization (Figure 1)^(15)^. These elements do not act in isolation but are in constant transformation. Relating the tetragram to the pregnancy-childbirth cycle of indigenous women allows us to recognize the complexity of this process in intercultural contexts and the need for expanded and inclusive health practices.

The following is a proposal for an approximation between the elements of the tetragram of complexity and interprofessional care in the pregnancy-puerperal cycle of indigenous women, recognizing the limits of this analysis and its partial, open, and ongoing nature, as required by complex thinking itself.

### Order

The order refers to established patterns, cultural norms, and rituals that guide social behavior. Among indigenous peoples, pregnancy, childbirth, and the postpartum period are guided by traditional knowledge, ancestral customs, well-defined collective functions, and rituals structured according to each ethnic group. This order promotes community cohesion, belonging, and symbolic security for pregnant women, who recognize themselves as part of a larger cycle^(19,20)^.

In the biomedical model, however, order is represented by standardized protocols, care flows, and technical standards, which are often disconnected from the subjectivities and sociocultural realities of indigenous women. While biomedical logic values regularity and predictability, indigenous knowledge conceives of health and disease as relational experiences involving the body, territory, community, ancestors, and spiritual forces^(21)^.

In the context of the Tetragrammaton element “order,” the National Policy for Indigenous Peoples’ Health Care (PNASPI) recognizes, albeit in a limited way, the need to articulate traditional practices and biomedical knowledge, proposing a model of differentiated and intercultural care. However, its implementation faces numerous challenges, such as the persistence of institutional ethnocentrism and the fragmentation of care practices^(22)^. Implemented by the Special Secretariat for Indigenous Health (SESAI), this policy establishes a differentiated care model with the aim of promoting, protecting, and restoring health, ensuring access to the SUS and the coordination of services close to communities^(23,24)^.

The effectiveness of PNASPI requires consideration of the cultural, geographic, and political specificities of each people, as well as the incorporation of appropriate technologies. The Special Indigenous Health Districts (DSEI), currently numbering 34 units, are the backbone of this care, integrating with the SUS through multidisciplinary teams involving health professionals, educators, and anthropologists. Noteworthy in this arrangement are the indigenous health agents and nurses, who work directly in the villages, coordinating care with itinerant teams^(22,23)^.

The care network includes base centers, SUS services, and Indigenous Health Houses (CASAI), organized to ensure comprehensive care at all levels of complexity. This model values cultural practices, the presence of family members, interpreters, and indigenous therapists, such as midwives, consolidating itself as a space for intercultural and interprofessional care^(22,23)^. Recent studies show that despite the existence of comprehensive and constructive policies, concrete actions are limited by various types of barriers, including structural, logistical, and communication barriers, among others^(11)^.

The experiences of indigenous women during pregnancy and childbirth are unique, rooted in cultural, environmental, and spiritual values that transcend scientific knowledge and challenge the linearity of conventional practices^(1)^. In light of this complexity, such experiences highlight the need to recognize the interdependence between different types of knowledge, integrating biological, social, cultural, and spiritual dimensions into a pluralistic perspective^(25)^.

The idea of order demands dialogue with the idea of disorder; the enriched idea of order draws on the ideas of interaction and organization, without excluding disorder^(15)^.

### Disorder

Disorder represents tensions, conflicts, and ruptures. The encounter between Western medicine and indigenous knowledge often generates clashes of reference, causing intercultural discomfort and tensions. The compulsory transfer of pregnant women to distant urban centers illustrates this disorder, as it breaks ties with care rituals and causes emotional suffering. However, according to complexity theory, disorder is also a creative force, opening up possibilities for more inclusive and equitable reorganization^(15,25)^.

In the context of care for indigenous women, disorder also manifests itself in professionals’ misunderstanding of traditional knowledge, the violation of reproductive rights, and submission to standardized hospital routines that ignore the desires and cultural practices of pregnant women. These situations generate insecurity, suffering, symbolic disconnection, and, often, lack of care^(11)^.

It is necessary to learn to think about order and disorder together, understanding that order is relative and relational, while disorder is marked by uncertainty^(15)^. Public health policies aimed at indigenous populations may be related to a need that disorder has awakened, making it necessary to think about more welcoming and less disruptive environments for these communities when they need care outside their villages. Therefore, disorder, in light of complexity, is not synonymous with destructive chaos, but represents the breaking of patterns, the emergence of the new and the unpredictable that destabilizes consolidated structures. Disorder, by disrupting the existing order, creates opportunities for the emergence of other more complex configurations^(15)^.

Therefore, disorder can also be productive. When health professionals encounter unforeseen situations, such as unfamiliar rituals, community practices not covered by protocols, or family decisions that escape institutional logic, there is an opportunity for listening and learning. This openness to the unexpected can challenge fragmented practices and create more respectful and responsive spaces for care^(15)^.

In the interprofessional field, disorder manifests itself in tensions between disciplinary knowledge, conflicts between care models, and communication barriers within teams. When not denied, these instabilities can foster transformative dialogues, driving the co-construction of more pluralistic and contextually rooted strategies.

### Interaction

Interaction is at the core of the tetragram, linking different elements, in this case traditional and biomedical practices. This link can take on conflicting or cooperative forms, depending on the willingness to engage in dialogue, active listening, and recognition of plural knowledge. In the pregnancy-puerperal cycle, experiences of humanized births in villages, involving midwives, indigenous health agents, and multidisciplinary teams, demonstrate that interaction can transform disorder into opportunity, reorganizing interprofessional care in a culturally respectful manner^(26,27)^.

Interaction is also observed in the encounter between indigenous pregnant women and health institutions. When this encounter is marked by institutional rigidity and the denial of cultural specificities, interactions become a source of conflict and exclusion. However, when there is cultural mediation, coordination with indigenous leaders, and institutional flexibility, possibilities open up for more humane, negotiated, and shared care. Thus, culturally sensitive public policies become essential, ensuring interculturality and the active participation of indigenous peoples in all stages of planning and execution, working together with differences^(13,18)^.

In the field of professional training, interaction between different areas of knowledge can contribute to strengthening care practices, provided that power asymmetries, institutional prejudices, and the need to build a common language are recognized, without disregarding singularities. Interprofessional care goes beyond a work organization technique; it is a comprehensive and ecological way of thinking and acting, capable of integrating dimensions of knowledge and practice in a transformative practice movement, with conceptual, methodological, and political implications directly related to the development of the field of health and education^(5)^.

Interprofessionality, characterized by mutual learning and integrated teamwork, is crucial to overcoming the fragmentation of care and promoting more comprehensive and effective health care, in line with the principles of the Unified Health System (SUS)^(4)^. Studies in Brazil and the Region indicate that cultural adaptations of prenatal care are associated with better care processes and reinforce strategies to accelerate the reduction of maternal mortality based on primary health care and cultural respect^(28)^.

In the field of health, interprofessional work has proven to be a way to overcome the fragmentation typical of the hegemonic biomedical model, favoring collaborative, safe, and integrated practices. This perspective strengthens ties between teams and communities, increases reciprocity among professionals, and enables collectively planned, contextualized, and evaluated actions oriented toward comprehensive care. However, the challenge remains of addressing historical inequalities and hierarchies that still structure the fields of health and education under colonized logics^(4,5)^.

In this scenario, interprofessionality and interdisciplinary training can take on an intercultural and emancipatory character, especially when applied to indigenous health in the pregnancy-puerperal cycle. Inspired by complexity theory, this approach recognizes the inseparability between order and disorder, technique and tradition, science and ancestry, opening space for more sensitive, dialogical, and transformative care practices^(5,10)^.

### Organization

From the dynamics between order, disorder, and interaction emerges organization, which translates into the construction of new care arrangements that are more inclusive and adapted to the reality of communities. This reorganization is not linear, but rather the result of constant negotiations, incorporating elements of interculturality and interprofessionality as strategies for strengthening comprehensive care^(13,16)^. In the pregnancy-puerperal cycle, this organization is expressed in care models that recognize indigenous women as protagonists, articulating technical and traditional knowledge to ensure culturally safe and socially just practices.

(Re)organization, as the fourth element of the tetragram, is the provisional result of interactions between order, disorder, and interaction. Instead of representing absolute stability, organization in complexity theory is transitory, adaptive, and constantly under reconstruction^(16)^. In indigenous women’s health care, the forms of health work organization must reflect this dynamic. Interprofessional teams need to act in a flexible, collaborative, and sensitive manner to local singularities, overcoming hierarchical and technocratic models that still predominate in many services, as well as the need for practices committed to the sustainable use of natural resources and understanding the interrelationships between social, environmental, and health factors^(13,29)^.

**Figure 2:**
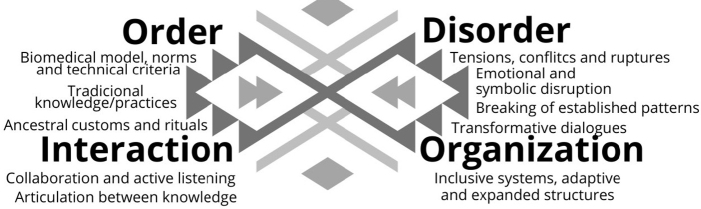
Representation of Morin’s tetragram integrated into the pregnancy-childbirth cycle of indigenous women.

Source: authors, 2025.

Therefore, a (re)organization of care based on complexity implies recognizing indigenous women as active participants in the process, integrating their choices, knowledge, and experiences into the pregnancy and postpartum cycle. It also involves strengthening community support networks, dialogue with midwives and traditional leaders, and valuing the territory as a space that promotes health. The construction of a relational organization of care also requires coordination between public policies, interprofessional strategies, and cultural mechanisms specific to each context^(30)^. It is an unfinished process that requires a willingness to deal with uncertainties, learn from mistakes, and continuously reorganize practices, as shown in Figure 2.

## FINAL CONSIDERATIONS

In light of complexity theory, this study allowed us to understand that the dynamics of interprofessional healthcare for indigenous women during pregnancy and childbirth are relational, situational, and non-linear processes marked by cultural, spiritual, social, and biological dimensions. The reflection showed that this care is built on the interaction between different types of knowledge, professional practices, and community arrangements, requiring approaches that go beyond fragmented and prescriptive models. Integrating complexity theory into indigenous health in the pregnancy-puerperal cycle offers a critical and integrative lens for understanding the multiple dimensions that traverse maternal and child health. Recognizing the interconnection between cultural, spiritual, social, and biological factors allows for the construction of health practices that respect and integrate traditional knowledge, promoting more equitable and effective care. The articulation between order, disorder, interaction, and organization allows us to understand this care as a dynamic, situated, and deeply relational phenomenon that requires openness to plurality, ethical sensitivity, and a willingness to reinvent practices in the face of everyday complexity.

From a professional practice perspective, it is important to strengthen interprofessional care strategies based on listening skills, recognition of community authorities, and coordination between different knowledge systems. For health training, there is a need for investment in intercultural and interprofessional training processes that incorporate complex thinking as a pedagogical tool, favoring the training of professionals who are sensitive to the ethical, political, and cultural dimensions of care in indigenous contexts. In the context of public policy, the study reinforces the urgency of planning and implementing actions that go beyond biomedical models focused exclusively on protocols and indicators, advancing in the construction of culturally safe, territorialized policies agreed upon with communities. Overcoming these challenges requires not only adequate public policies, but also intercultural training for health professionals, in order to ensure that indigenous women have access to care that respects their identity, promotes well-being, and strengthens community ties.

However, we recognize the epistemological limitations inherent in the theoretical-reflective method adopted. The absence of a systematic search of the literature and the lack of direct empirical dialogue with concrete experiences of care may restrict the scope of the reflections, which are predominantly conceptual and interpretative in nature. The immense cultural and social diversity of indigenous peoples in Brazil constitutes a limitation to the reflections presented in this study. The tensions and possibilities of interprofessional care vary widely between different ethnic groups and regions, and this reflection cannot be generalized to all intercultural contexts. The contributions of this study to the advancement of nursing science are related to the proposition of a complex thought that makes it possible to unveil the encounters, tensions, and possibilities present in the interprofessional care of indigenous women.

## DATA AVAILABILITY

The entire dataset supporting the results of this study was published in the article itself.

## References

[1] Boer L, Sousa FGM, Pina RMP, Poblete M, Haeffner LSB, Backes DS (2024). Indigenous women’s experiences about the pregnancy-puerperal
cycle.. Rev Bras Enferm.

[2] Backes DS, Morais TR, Rosa CB, Haeffner LSB, Galvão DMPG, Pereira AD (2025). Care for women in the pregnancy-puerperium cycle from the
perspective of health professionals in the light of complexity
thinking.. Rev Lat Am Enfermagem.

[3] Backes DS, Zinhani MC, Erdmann AL, Backes MTS, Büscher A, Caino Marchiori MRT (2022). Nursing care as a systemic and entrepreneurial
phenomenon.. Rev Esc Enferm USP.

[4] Rosa OM, Frizon G, Almeida MCV, Krummenauer R, Backes MS, Backes DS (2022). Educação interprofissional em saúde: elucidando
conceitos.. Res Soci Dev.

[5] Pereira MF (2018). Interprofissionalidade e saúde: conexões e fronteiras em
transformação.. Interface.

[6] Organização Pan-Americana da Saúde. (2025). Interprofessional health teams for integrated care..

[7] Brasil. Ministério da Ciência, Tecnologia e Inovação. (2023). Diário Oficial da União.

[8] Fiocruz. (2022). Determinantes sociais da saúde [Internet].. https://pensesus.fiocruz.br/determinantes-sociais.

[9] Commission on Social Determinants of Health. (2008). Closing the gap in a generation: health equity through action on the
social determinants of health..

[10] Morin E (2003). Introdução ao pensamento complexo..

[11] Almeida RM, Paes FT, Gomes AS, Lacerda RGF, Way JRSW, Ribeiro NL (2025). Saúde, educação e direitos dos povos indígenas e das comunidades
tradicionais, sob a lente das organizações nacionais e
internacionais.. Rev Saúde Redes.

[12] Alves HJ, Soares MRP, Costa RRS, Santos LA (2023). Saúde da Família, territórios quilombolas e a defesa da
vida.. Trab Educ Saúde.

[13] Honorato MM, Oliveira NP, Domingues RJS, Cremaschi RMC, Coelho FMS, Silva JAC (2022). Princípio bioético da autonomia na atenção à saúde
indígena.. Rev Bioet.

[14] Petraglia I (2013). Pensamento complexo e educação..

[15] Morin E (2024). Ciência com consciência..

[16] Morin E (2006). A cabeça bem-feita: repensar a reforma, reformar o pensamento..

[17] Shepherd SM, Delgado RH, Sherwood J, Paradies Y (2018). The impact of indigenous cultural identity and cultural
engagement on violent offending.. BMC Public Health.

[18] Macedo V (2021). O cuidado e suas redes: doença e diferença em instituições de
saúde indígena em São Paulo.. Rev Bras Cienc Soc.

[19] Rodrigues DP, Alves VH, Silva AM, Penna LHG, Vieira BDG, Silva SÉD (2022). Women’s perception of labor and birth care: obstacles to
humanization.. Rev Bras Enferm.

[20] Lima CMA, Ribeiro KA, Santos JGC (2018). Iniciação sexual, gestação, parto e puerpério em comunidades
indígenas do Brasil: uma breve revisão integrativa.. Rev Saúde Pública Mato Grosso do Sul.

[21] Barreto JPL (2017). Bahserikowi – Centro de Medicina Indígena da
Amazônia.. Amazônica.

[22] Brasil. Ministério da Saúde. (2025). Secretaria Especial de Saúde Indígena. Estrutura – Distritos
Sanitários Especiais Indígenas (DSEI) [Internet].. https://www.gov.br/saude/pt-br/composicao/sesai/estrutura/dsei.

[23] Brasil. Fundação Nacional de Saúde. (2002). Política nacional de atenção à saúde dos povos indígenas..

[24] Brasil. Decreto nº 9.795, de 17 de maio de 2019. (2019). Diário Oficial da União.

[25] Melo AV, Sant’Ana GR, Bastos PRHO, Antônio L (2021). Bioética e interculturalidade na atenção à saúde
indígena.. Rev Bioet.

[26] Santos WP, Moraes IS, Rodrigues MPS (2024). Diálogos decoloniais sobre o parir: a experiência das oficinas de
trocas de saberes com parteiras tradicionais do Amazonas.. Rev Saúde Redes.

[27] Casagranda F, Luz VG, Martins CP, Dias-Scopel RP, Fernandes R, Fonseca W (2024). A saúde indígena na atenção especializada: perspectiva dos
profissionais de saúde em um hospital de referência no Mato Grosso do Sul,
Brasil.. Cad Saude Publica.

[28] Abreu GR, Picoli RP, Welch JR, Coimbra CEA (2024). Adequação da assistência pré-natal ofertada à mulher indígena:
características maternas e dos serviços de saúde.. Cien Saude Colet.

[29] Backes DS, Halmenschlager RR, Cassola TP, Erdmann AL, Hämel K, Costenaro RGS (2024). Inseparability between public health, planetary health and the
nursing process: premise for sustainable development.. Rev Esc Enferm USP.

[30] Lima LGA, Ricarte EC, Aguiar MSS, Lima AS, Figueiredo SD, Lima SHN (2025). A importância do diálogo intercultural na humanização da
assistência à saúde indígena.. Saude Colet.

