# Supplementary material for: Interprofessional care for Indigenous women during pregnancy and childbirth in light of Complexity Theory
Source: Rev Esc Enferm USP. 2026 May 11;60:e20250504. doi: 10.1590/1980-220X-REEUSP-2025-0504en (PMC13166080; doi:10.1590/1980-220X-REEUSP-2025-0504en)
Supplement: Translation

# Translation: Cuidado interprofissional de mulheres indígenas no ciclo gravídico-puerperal à luz da Teoria da Complexidade

**DOI:** 10.1590/1980-220X-REEUSP-2025-0504pt

**Authors:** Julieli Rosso, Luana Pizarro Meneghello, Silvia Naujorks, Janet Mercedes Arévalo-Ipanaqué, Dirce Stein Backes, Juliana Silveira Colomé

**Affiliations:** 1 Universidade Franciscana, Programa de Pós-Graduação em Saúde Materno-Infantil, Santa Maria, RS, Brasil.; 2 Universidad Peruana Cayetano Heredia, Facultad de Enfermería, Lima, Peru.

**Keywords:** Saúde de Populações Indígenas, Assistência Perinatal, Saúde Materno-Infantil, Relações Interprofissionais, Filosofia

## Abstract

**Objetivo:**

Refletir, à luz da teoria da complexidade, as dinâmicas do cuidado interprofissional à saúde das mulheres indígenas no ciclo gravídico-puerperal.

**Método:**

Estudo teórico-reflexivo, empregando o referencial da complexidade de Edgar Morin, por meio do tetragrama para desvelar os encontros, tensões e possibilidades presentes no cuidado interprofissional em contextos interculturais.

**Resultados:**

O cuidado interprofissional emerge como estratégia crucial para superar a fragmentação do modelo biomédico hegemônico. A aplicação do tetragrama demonstrou que: a ordem reside nas práticas tradicionais e protocolos biomédicos; a desordem manifesta-se nas rupturas e conflitos culturais; a interação ocorre nas experiências de articulação entre saberes; e a organização indica a construção de arranjos de cuidado mais inclusivos e adaptativos.

**Conclusão:**

A teoria da complexidade oferece uma lente fecunda para repensar e (re)orientar a atenção à saúde das mulheres indígenas, destacando a imperatividade da escuta ativa, valorização dos saberes tradicionais e construção de práticas interprofissionais culturalmente seguras, contextualizadas e transformadoras.

## INTRODUÇÃO

A saúde indígena no ciclo gravídico-puerperal constitui um campo que exige compreensão sistêmica, sensível e integrada das dimensões culturais, espirituais, sociais e biológicas^(1,2)^. Nesse contexto, faz-se necessário o fomento de práticas interprofissionais que superem a fragmentação e o isolamento entre as categorias profissionais. Tais abordagens exigem o desenvolvimento de uma liderança compartilhada e evolutiva, capaz de articular e promover a cooperação necessária para responder de forma eficaz à complexidade dos cuidados de saúde^(3,4)^.

O cuidado interprofissional na saúde e na educação configura-se como um eixo estratégico para a construção de arranjos formativos e práticos que articulam saberes distintos em processos de experimentação, cooperação e produção coletiva. Equipes interprofissionais são fundamentais na melhoria da capacidade dos sistemas de saúde no atendimento às necessidades de saúde da população^(5,6)^.

Estudos denotam que mulheres indígenas no Brasil apresentam maiores taxas de morbimortalidade materna e infantil quando comparadas à população geral, influenciadas por determinantes sociais como pobreza, exclusão social, disparidades educacionais e raciais, barreiras geográficas e discriminação institucional^(7,8,9)^. Diante da natureza multifacetada dessas desigualdades, faz-se necessário um referencial teórico capaz de integrar múltiplas dimensões.

Considerando a interrelação entre fatores culturais, sociais, institucionais e espirituais, torna-se evidente que abordagens lineares não dão conta da complexidade envolvida no cuidado à saúde das mulheres indígenas. Ademais, apesar dos avanços nas políticas públicas, há escassez de estudos que abordem o cuidado interprofissional à saúde das mulheres indígenas a partir da perspectiva da complexidade.

A teoria da complexidade, proposta por Edgar Morin, oferece uma abordagem robusta para refletir criticamente sobre o cuidado interprofissional à saúde das mulheres indígenas, superando visões fragmentadas e lineares que predominam nos modelos biomédicos tradicionais. A complexidade é um tecido de constituintes heterogêneos inseparavelmente associados, o que desafia a dicotomia entre o uno e o múltiplo e propõe uma visão que integra diversidade e interconexão^(10)^. O cuidado à saúde indígena exige o reconhecimento de diferentes concepções de vida, saúde e doença. Isso demanda uma postura ética que supere os limites do modelo biomédico universalista, frequentemente excludente dos saberes ancestrais^(11,12,13)^.

Argumenta-se que o referencial da complexidade pode sustentar uma análise sensível, crítica e integradora do cuidado interprofissional à saúde das mulheres indígenas no contexto intercultural. Assim, questiona-se: como a teoria da complexidade pode contribuir para a reflexão e para a prática do cuidado interprofissional à saúde das mulheres indígenas no ciclo gravídico-puerperal?”

Desse modo, este estudo teórico-reflexivo, ao dialogar com Edgar Morin, busca refletir, à luz da teoria da complexidade, as dinâmicas do cuidado interprofissional à saúde das mulheres indígenas no ciclo gravídico-puerperal.

## MÉTODO

Trata-se de um estudo teórico-reflexivo, de abordagem qualitativa, construído a partir do referencial do Pensamento Complexo de Edgar Morin, especialmente por meio do tetragrama Morin (ordem, desordem, interação e organização) como referencial analítico para desvelar os encontros, tensões e possibilidades presentes no cuidado interprofissional em contextos interculturais, no contexto da disciplina “Construção do Pensamento Sistêmico”, cursada no âmbito da formação doutoral. Concebido inicialmente como capítulo de livro, o texto foi reelaborado e adaptado ao formato de artigo científico-reflexivo.

O percurso fundamentou-se na articulação entre o referencial teórico central e um corpus de produções científicas nacionais e internacionais relacionadas ao cuidado interprofissional e à saúde indígena. A seleção dessas obras ocorreu de forma intencional e analítica, considerando sua preocupação temática, relevância conceitual e potencial de diálogo com os pressupostos do/a pensamento/teoria da complexidade/complexo.

As buscas bibliográficas foram realizadas na Biblioteca Virtual em Saúde (BVS), contemplando artigos, documentos técnicos e produções teóricas pertinentes ao campo da saúde coletiva, enfermagem e saúde indígena. Não se tratou de uma revisão sistemática, mas de um levantamento teórico orientado pela problematização reflexiva, buscando subsidiar a construção argumentativa e a análise crítica proposta.

A elaboração do estudo ocorreu no período de agosto a outubro de 2025, considerando publicações disponíveis até esse intervalo temporal, o que contribui para a contextualização e atualidade da reflexão desenvolvida. O processo analítico consistiu na leitura aprofundada, interpretação crítica e articulação conceitual das obras selecionadas, em diálogo contínuo com o referencial de Morin, buscando compreender a complexidade do cuidado em saúde em contextos marcados por interprofissionalidade e diversidade sociocultural.

Assim, o presente estudo delineia-se com base no arcabouço teórico-reflexivo da teoria da complexidade de Edgar Morin, a fim de fundamentar o cuidado interprofissional à saúde das mulheres indígenas no ciclo gravídico-puerperal.

### O Pensamento Complexo de Edgar Morin

Como já apresentado, o referencial adotado fundamenta-se na teoria da complexidade proposta por Edgar Morin, filósofo, sociólogo e epistemólogo francês, nascido em 1921. Sua biografia, marcada por perdas precoces, como a morte da mãe, aos nove anos, contribuiu para uma sensibilidade particular diante da condição humana e suas múltiplas dimensões. Ao longo de sua trajetória intelectual, Morin demonstrou interesse pela literatura, música e cinema, transitando por diferentes campos do conhecimento e consolidando uma visão integradora, sobretudo nas ciências humanas^(14)^.

A complexidade é um conjunto de elementos inseparavelmente associados, que se expressa como um tecido de acontecimentos, ações, interações, retroações, determinações e acasos que constituem a realidade^(15)^. Nesse entrelaçamento, estão presentes incertezas, indeterminações e aleatoriedades que desafiam visões reducionistas ou lineares^(16)^.

A teoria da complexidade busca integrar aspectos frequentemente vistos como contraditórios, unidade e diversidade, continuidade e ruptura, construção e desconstrução, reconhecendo que não é possível analisar as partes sem o todo, nem o todo sem as partes. Nesse sentido, a complexidade deve ser entendida como uma palavra-problema, e não como uma palavra-solução: não fornece respostas prontas, mas convida à reflexão permanente, à análise crítica e à abertura ao inesperado^(15)^.

À luz da teoria da complexidade, reflete-se a coexistência entre ordem e desordem, ilustrando sua ideia com a observação do céu estrelado. O que inicialmente parece um agrupamento aleatório de astros revela, com olhar atento, uma ordem cósmica; ao mesmo tempo, ao se considerar o universo em expansão, percebe-se a desordem criativa de estrelas que nascem, explodem e morrem. Assim, ordem e desordem constituem dimensões inseparáveis do real^(15)^.

Essa concepção é fundamental para pensar os desafios da atenção à saúde indígena no ciclo gravídico-puerperal, marcado por uma complexa tessitura de elementos culturais, espirituais, sociais e biológicos. O cuidado às mulheres indígenas não pode ser reduzido à lógica biomédica linear, baseada em protocolos técnicos, é preciso reconhecer a coexistência de múltiplas ordens e desordens, saberes tradicionais e científicos, rotinas institucionais e práticas ancestrais, direitos conquistados e violências históricas ainda presentes^(1,17)^.

A teoria da complexidade se sustenta em princípios que se articulam no circuito tetralógico ou tetragrama – ordem, desordem, interação e organização, orientando a reflexão para a religação dos saberes^(15)^. Os princípios da teoria da complexidade, propostos por Morin, oferecem bases essenciais para compreender fenômenos em sua totalidade. O princípio sistêmico mostra que partes e todo são indissociáveis, o hologramático revela que cada parte contém o todo e vice-versa, superando reducionismos e holismos. O circuito retroativo e o recursivo destacam a autorregulação e a circularidade entre causas e efeitos. A autonomia está sempre em relação de dependência com o meio, enquanto o princípio dialógico integra noções opostas que coexistem na realidade. Por fim, a reintrodução do conhecimento propõe uma reforma do pensamento, reconhecendo limites, incertezas e a necessidade de constante contextualização.

No campo da saúde indígena, os princípios da teoria da complexidade de Morin ajudam a compreender o cuidado de forma ampliada. O princípio dialógico evidencia a necessidade de integrar saberes técnicos e saberes tradicionais, permitindo que ciência e ancestralidade coexistam em diálogo. O princípio recursivo mostra que as práticas de cuidado não se esgotam em si mesmas, mas retroalimentam a cultura, a saúde e o território, fortalecendo continuamente a vida comunitária. Já o princípio hologramático revela que cada parte, seja o profissional, a comunidade, a mulher ou a família, contém e reflete o todo, mostrando que o cuidado é simultaneamente individual e coletivo, singular e comunitário^(16,18)^.

### O Tetragrama de Morin e o Ciclo Gravídico-Puerperal Indígena

À luz da teoria da complexidade, os processos dinâmicos da realidade podem ser compreendidos através do tetragrama, uma proposição que considera que qualquer sistema vivo ou social é resultante da intersecção entre ordem, desordem, interação e organização (Figura 1)^(15)^. Esses elementos não atuam isoladamente, mas em constante movimento de transformação. Relacionar o tetragrama ao ciclo gravídico-puerperal das mulheres indígenas permite reconhecer a complexidade desse processo em contextos interculturais e a necessidade de práticas de saúde ampliadas e inclusivas.

**Figura 1:** Tetragrama de Morin.

Fonte: Morin^(15:204)^.

Propõe-se, a seguir, uma aproximação entre os elementos do tetragrama da complexidade e o cuidado interprofissional no ciclo gravídico-puerperal de mulheres indígenas, reconhecendo os limites dessa análise e sua natureza parcial, aberta e em construção, como exige o próprio pensamento complexo.

### Ordem

A ordem refere-se a padrões estabelecidos, normas culturais e rituais que orientam o comportamento social. Entre povos indígenas, a gestação, o parto e o puerpério são conduzidos a partir de saberes tradicionais, costumes ancestrais, funções coletivas bem definidas e rituais estruturados de acordo com cada etnia. Essa ordem promove coesão comunitária, pertencimento e segurança simbólica para a gestante, que se reconhece como parte de um ciclo maior^(19,20)^.

Já no modelo biomédico, a ordem é representada por protocolos padronizados, fluxos assistenciais e normativas técnicas, muitas vezes desconectadas das subjetividades e realidades socioculturais das mulheres indígenas. Enquanto a lógica biomédica valoriza a regularidade e a previsibilidade, os saberes indígenas concebem a saúde e a doença como experiências relacionais, que envolvem o corpo, o território, a coletividade, os ancestrais e as forças espirituais^(21)^.

No contexto do elemento “ordem” do Tetragrama, a Política Nacional de Atenção à Saúde dos Povos Indígenas (PNASPI) reconhece, ainda que de forma limitada, a necessidade de articular práticas tradicionais e conhecimentos biomédicos, propondo um modelo de atenção diferenciada e intercultural. No entanto, sua implementação enfrenta inúmeros desafios, como a persistência do etnocentrismo institucional e a fragmentação das práticas de cuidado^(22)^. Executada pela Secretaria Especial de Saúde Indígena (SESAI), essa política estabelece um modelo de atenção diferenciada, com o objetivo de promover, proteger e recuperar a saúde, garantindo o acesso ao SUS e a articulação de serviços próximos às comunidades^(23,24)^.

A efetividade da PNASPI exige a consideração das especificidades culturais, geográficas e políticas de cada povo, bem como a incorporação de tecnologias apropriadas. Os Distritos Sanitários Especiais Indígenas (DSEI), atualmente em 34 unidades, são o eixo estruturante dessa atenção, integrando-se ao SUS por meio de equipes multiprofissionais que envolvem profissionais de saúde, educadores e antropólogos. Destacam-se, nesse arranjo, os agentes indígenas de saúde e enfermeiros, que atuam diretamente nas aldeias, articulando o cuidado com equipes itinerantes^(22,23)^.

A rede de atenção inclui polos-base, serviços do SUS e Casas de Saúde Indígena (CASAI), organizando-se para garantir cuidado integral em todos os níveis de complexidade. Esse modelo valoriza práticas culturais, a presença de familiares, intérpretes e terapeutas indígenas, como as parteiras, consolidando-se como espaço de cuidado intercultural e interprofissional^(22,23)^. Estudos recentes demonstram que apesar da existência de políticas abrangentes e construtivas, as ações concretas se tornam limitadas por diversos tipos de barreiras, estruturais, logísticas, de comunicação, entre outras^(11)^.

As experiências das mulheres indígenas no ciclo gravídico-puerperal apresentam singularidades enraizadas em valores culturais, ambientais e espirituais, que por sua profundidade transcendem o conhecimento científico e questionam a linearidade das práticas convencionais^(1)^. À luz da complexidade, tais vivências evidenciam a necessidade de reconhecer a interdependência entre saberes distintos, integrando dimensões biológicas, sociais, culturais e espirituais em uma perspectiva plural^(25)^.

A ideia de ordem demanda o diálogo com a ideia de desordem, a ideia enriquecida de ordem recorre às ideias de interação e de organização, sem excluir a desordem^(15)^.

### Desordem

A desordem representa tensões, conflitos e rupturas. O encontro entre a medicina ocidental e os saberes indígenas frequentemente gera choques de referência, provocando desconfortos e tensões interculturais. A transferência compulsória de gestantes para centros urbanos distantes ilustra essa desordem, pois rompe vínculos com rituais de cuidado e gera sofrimento emocional. Entretanto, segundo a teoria da complexidade, a desordem também é potência criativa, abrindo possibilidades de reorganização mais inclusivas e equânimes^(15,25)^.

No contexto do cuidado às mulheres indígenas, a desordem também se manifesta na incompreensão dos saberes tradicionais pelos profissionais, na violação de direitos reprodutivos e na submissão a rotinas hospitalares padronizadas que ignoram os desejos e práticas culturais das gestantes. Essas situações geram insegurança, sofrimento, desconexão simbólica e, muitas vezes, desassistência^(11)^.

Faz-se necessário aprender a pensar ordem e desordem conjuntamente, entendendo que a ordem é relativa e relacional, enquanto a desordem é marcada pela incerteza^(15)^. As políticas públicas de saúde voltadas às populações indígenas podem estar relacionadas a uma necessidade em que a desordem despertou, sendo necessário pensar em ambientes mais acolhedores e menos ruptivos para essas comunidades quando necessitam de atendimento fora das aldeias. Portanto, a desordem, à luz da complexidade, não é sinônimo de caos destrutivo, mas representa o rompimento de padrões, o surgimento do novo e o imprevisível que desestabiliza estruturas consolidadas. A desordem, ao perturbar a ordem existente, cria oportunidades para a emergência de outras configurações mais complexas^(15)^.

Portanto, a desordem também pode ser produtiva. Quando os profissionais de saúde se deparam com situações imprevistas, por exemplo, rituais desconhecidos, práticas comunitárias não previstas nos protocolos ou decisões familiares que escapam às lógicas institucionais, há a possibilidade de escuta e aprendizado. Essa abertura ao inesperado pode tensionar práticas fragmentadas e gerar espaços de cuidado mais respeitosos e responsivos^(15)^.

No campo interprofissional, a desordem se expressa nas tensões entre saberes disciplinares, nos conflitos entre modelos de atenção e nos limites comunicacionais das equipes. Essas instabilidades, quando não negadas, podem fomentar diálogos transformadores, impulsionando a co-construção de estratégias mais plurais e contextualmente enraizadas.

### Interação

A interação constitui o núcleo do tetragrama, articulando elementos distintos, neste caso, práticas tradicionais e biomédicas. Essa articulação pode assumir formas conflituosas ou cooperativas, dependendo da disposição para o diálogo, da escuta ativa e do reconhecimento de saberes plurais. No ciclo gravídico-puerperal, experiências de partos humanizados em aldeias, envolvendo parteiras, agentes indígenas de saúde e equipes multiprofissionais demonstram que a interação pode transformar a desordem em oportunidade, reorganizando o cuidado interprofissional de forma culturalmente respeitosa^(26,27)^.

A interação também se observa no encontro entre as gestantes indígenas e as instituições de saúde. Quando esse encontro é marcado pela rigidez institucional e pela negação das especificidades culturais, as interações tornam-se fonte de conflito e exclusão. Porém, quando há mediação cultural, articulação com lideranças indígenas e flexibilidade institucional, abrem-se possibilidades para um cuidado mais humano, negociado e compartilhado. Assim, políticas públicas culturalmente sensíveis tornam-se imprescindíveis, garantindo interculturalidade e participação ativa dos povos indígenas em todas as etapas de planejamento e execução, trabalhando juntamente com as diferenças^(13,18)^.

No campo da formação profissional, a interação entre diferentes áreas do saber pode contribuir para o fortalecimento das práticas de cuidado, desde que se reconheçam as assimetrias de poder, os preconceitos institucionais e a necessidade de construir uma linguagem comum, sem desconsiderar as singularidades. O cuidado interprofissional vai além de uma técnica de organização do trabalho, trata-se de uma forma integral e ecológica de pensar e agir, capaz de integrar dimensões do conhecimento e da prática em um movimento de prática transformadora, com implicações conceituais, metodológicas e políticas diretamente relacionadas ao desenvolvimento do campo da saúde e da educação^(5)^.

A interprofissionalidade, caracterizada pela aprendizagem mútua e trabalho em equipe integrado, é crucial para superar a fragmentação do cuidado e promover uma atenção à saúde mais abrangente e eficaz, alinhada aos princípios do Sistema Único de Saúde (SUS)^(4)^. Estudos no Brasil e na Região apontam que adaptações culturais do pré-natal estão associadas a melhor processo de cuidado, reforçam estratégias para acelerar a redução da mortalidade materna baseadas na atenção primária de saúde e no respeito cultural^(28)^.

No campo da saúde, o trabalho interprofissional revela-se como uma via para superar a fragmentação típica do modelo biomédico hegemônico, favorecendo práticas colaborativas, seguras e integradas. Essa perspectiva fortalece vínculos entre equipes e comunidades, amplia a reciprocidade entre profissionais e possibilita ações coletivamente planejadas, contextualizadas e avaliadas, orientadas para o cuidado integral. Contudo, persiste o desafio de enfrentar desigualdades e hierarquias históricas que ainda estruturam os campos da saúde e da educação sob lógicas colonizadas^(4,5)^.

Nesse cenário, a interprofissionalidade e a formação interdisciplinar podem assumir um caráter intercultural e emancipatório, sobretudo quando aplicadas à saúde indígena no ciclo gravídicopuerperal. Inspirada na teoria da complexidade, essa abordagem reconhece a inseparabilidade entre ordem e desordem, técnica e tradição, ciência e ancestralidade, abrindo espaço para práticas de cuidado mais sensíveis, dialógicas e transformadoras^(5,10)^.

### Organização

Da dinâmica entre ordem, desordem e interação emerge a organização, que traduz a construção de novos arranjos de cuidado, mais inclusivos e adaptados à realidade das comunidades. Essa reorganização não é linear, mas fruto de negociações constantes, incorporando elementos da interculturalidade e da interprofissionalidade como estratégias de fortalecimento do cuidado integral^(13,16)^. No ciclo gravídico-puerperal, essa organização se expressa em modelos de atenção que reconhecem a mulher indígena como protagonista, articulando saberes técnicos e tradicionais para assegurar práticas culturalmente seguras e socialmente justas.

A (re)organização, como quarto elemento do tetragrama, é o resultado provisório das interações entre ordem, desordem e interação. Em vez de representar estabilidade absoluta, a organização na teoria da complexidade é transitória, adaptativa e em constante reconstrução^(16)^. No cuidado à saúde das mulheres indígenas, as formas de organização do trabalho em saúde devem refletir essa dinâmica. Equipes interprofissionais precisam atuar de maneira flexível, colaborativa e sensível às singularidades locais, superando modelos hierárquicos e tecnocráticos que ainda predominam em muitos serviços, bem como a necessidade de práticas comprometidas com o uso sustentável dos recursos naturais e com a compreensão das inter-relações entre fatores sociais, ambientais e de saúde^(13,29)^.

Portanto, uma (re)organização do cuidado baseada na complexidade implica reconhecer a mulher indígena como sujeito ativo do processo, integrando suas escolhas, saberes e experiências no ciclo gravídico-puerperal. Envolve, ainda, o fortalecimento de redes de apoio comunitário, o diálogo com parteiras e lideranças tradicionais, e a valorização do território como espaço produtor de saúde. A construção de uma organização relacional do cuidado demanda, ainda, a articulação entre políticas públicas, estratégias interprofissionais e dispositivos culturais específicos de cada contexto^(30)^. Trata-se de um processo inacabado, que requer disposição para lidar com incertezas, aprender com os erros e reorganizar continuamente as práticas, conforme demonstrado na Figura 2.

**Figura 2:** Representação do Tetragrama de Morin integrado ao ciclo gravídico-puerperal de mulheres indígenas.

Fonte: autores, 2025.

## CONSIDERAÇÕES FINAIS

À luz da teoria da complexidade, este estudo permitiu compreender que as dinâmicas do cuidado interprofissional à saúde das mulheres indígenas no ciclo gravídico-puerperal se configuram como processos relacionais, situados e não lineares, marcados por dimensões culturais, espirituais, sociais e biológicas. A reflexão evidenciou que esse cuidado se constrói na interação entre diferentes saberes, práticas profissionais e arranjos comunitários, exigindo abordagens que superem modelos fragmentados e prescritivos. Integrar a teoria da complexidade à saúde indígena no ciclo gravídico-puerperal oferece uma lente crítica e integradora para compreender as múltiplas dimensões que atravessam a saúde materno-infantil. Reconhecer a interconexão entre fatores culturais, espirituais, sociais e biológicos permite construir práticas de saúde que respeitem e integrem saberes tradicionais, promovendo atenção mais equitativa e eficaz. A articulação entre ordem, desordem, interação e organização permite compreender esse cuidado como um fenômeno dinâmico, situado e profundamente relacional, que exige abertura à pluralidade, sensibilidade ética e disposição para reinventar práticas diante da complexidade cotidiana.

Do ponto de vista da prática profissional, sugere-se a importância de fortalecer estratégias de cuidado interprofissional com base na escuta de habilidades, no reconhecimento das autoridades comunitárias e na articulação entre diferentes sistemas de conhecimento. Para a formação em saúde, destaca-se a necessidade de investimentos em processos formativos interculturais e interprofissionais, que incorporam o pensamento complexo como ferramenta pedagógica, favorecendo a formação de profissionais sensíveis às dimensões éticas, políticas e culturais do cuidado em contextos indígenas. No âmbito das políticas públicas, o estudo reforça a urgência de planejar e implementar ações que ultrapassem modelos biomédicos centrados exclusivamente em protocolos e indicadores, avançando na construção de políticas culturalmente seguras, territorializadas e pactuadas com as comunidades. A superação dos desafios requer não apenas políticas públicas adequadas, mas também formação intercultural para profissionais de saúde, de modo a assegurar que as mulheres indígenas tenham acesso a cuidados que respeitem sua identidade, promovam bem-estar e fortaleçam os vínculos comunitários.

Reconhece-se, contudo, as limitações epistemológicas inerentes ao método teórico-reflexivo adotado. A ausência de uma busca sistemática na literatura e a inexistência de diálogo empírico direto com experiências concretas de cuidado podem restringir o alcance das reflexões, que se sustentam predominantemente no plano conceitual e interpretativo. A imensa diversidade cultural e social dos povos indígenas no Brasil constitui-se em limitação para as reflexões apresentadas neste estudo. As tensões e possibilidades do cuidado interprofissional variam amplamente entre diferentes etnias e regiões, e esta reflexão não pode ser generalizada para a totalidade dos contextos interculturais. As contribuições deste estudo para o avanço da ciência de enfermagem estão relacionadas à proposição de um pensamento complexo que possibilita desvelar os encontros, tensões e possibilidades presentes no cuidado interprofissional de mulheres indígenas.

## DISPONIBILIDADE DE DADOS

Todo o conjunto de dados que dá suporte aos resultados deste estudo foi publicado no próprio artigo.

# Translation: Cuidado interprofesional de mujeres indígenas durante el ciclo grávido-puerperal a la luz de la Teoría de la Complejidad

**DOI:** 10.1590/1980-220X-REEUSP-2025-0504es

**Authors:** Julieli Rosso, Luana Pizarro Meneghello, Silvia Naujorks, Janet Mercedes Arévalo-Ipanaqué, Dirce Stein Backes, Juliana Silveira Colomé

**Affiliations:** 1 Universidade Franciscana, Programa de Pós-Graduação em Saúde Materno-Infantil, Santa Maria, RS, Brasil.; 2 Universidad Peruana Cayetano Heredia, Facultad de Enfermería, Lima, Peru.

**Keywords:** Salud de Poblaciones Indígenas, Atención Perinatal, Salud Materno-Infantil, Relaciones Interprofesionales, Filosofía.

## Abstract

**Objetivo:**

Reflexionar sobre las dinámicas del cuidado interprofesional de la salud de mujeres indígenas durante el ciclo grávido-puerperal, a la luz de la teoría de la complejidad.

**Método:**

Es un estudio teórico-reflexivo llevado a cabo mediante tetragrama con el referencial de la complejidad de Edgar Morin, para revelar encuentros, tensiones y posibilidades de la atención interprofesional en contextos interculturales.

**Resultados:**

La atención interprofesional surge como una estrategia crucial para superar la fragmentación del modelo biomédico hegemónico. La aplicación del tetragrama demostró que: el orden reside en las prácticas tradicionales y protocolos biomédicos; el desorden asoma en las rupturas y conflictos culturales; la interacción se produce en las experiencias de articulación entre saberes; y la organización señala la construcción de una atención más inclusiva y adaptativa.

**Conclusión:**

La teoría de la complejidad ofrece una perspectiva fructífera para repensar y (re)orientar la atención a la salud de mujeres indígenas, destacando la importancia de la escucha activa, la valorización de los conocimientos tradicionales y la construcción de prácticas interprofesionales culturalmente seguras, contextualizadas y transformadoras.

## INTRODUCCIÓN

La salud indígena durante el ciclo grávido-puerperal constituye un ámbito que exige una comprensión sistémica, sensible e integrada de las dimensiones culturales, espirituales, sociales y biológicas^(1,2)^. En este contexto, es necesario fomentar prácticas interprofesionales que superen la fragmentación y el aislamiento entre las categorías profesionales. Tales enfoques exigen el desarrollo de un liderazgo compartido y evolutivo, capaz de articular y promover la cooperación necesaria para responder de manera eficaz a la complejidad de la atención de salud^(3,4)^.

La atención interprofesional en salud y educación se configura como un eje estratégico para la construcción de arreglos formativos y prácticos que articulan conocimientos distintos en procesos de experimentación, cooperación y producción colectiva. Los equipos interprofesionales son fundamentales para mejorar la capacidad de los sistemas de salud en la atención de las necesidades de salud de la población^(5,6)^.

Los estudios indican que las mujeres indígenas en Brasil presentan mayores tasas de morbilidad y mortalidad materna e infantil en comparación con la población general, influenciadas por determinantes sociales como la pobreza, la exclusión social, las disparidades educativas y raciales, las barreras geográficas y la discriminación institucional^(7,8,9)^. Ante la naturaleza multifacética de estas desigualdades, se hace necesario un marco teórico capaz de integrar múltiples dimensiones.

Teniendo en cuenta la interrelación entre factores culturales, sociales, institucionales y espirituales, resulta evidente que los enfoques lineales no dan cuenta de la complejidad que implica la atención de la salud de las mujeres indígenas. Además, a pesar de los avances en las políticas públicas, hay una escasez de estudios que aborden la atención interprofesional de la salud de las mujeres indígenas desde la perspectiva de la complejidad.

La teoría de la complejidad, propuesta por Edgar Morin, ofrece un enfoque sólido para reflexionar críticamente sobre la atención interprofesional de la salud de las mujeres indígenas, superando las visiones fragmentadas y lineales que predominan en los modelos biomédicos tradicionales. La complejidad es un tejido de componentes heterogéneos inseparablemente asociados, lo que desafía la dicotomía entre lo uno y lo múltiple y propone una visión que integra la diversidad y la interconexión^(10)^. La atención de la salud indígena exige el reconocimiento de diferentes concepciones de la vida, la salud y la enfermedad. Esto requiere una postura ética que supere los límites del modelo biomédico universalista, que con frecuencia excluye los saberes ancestrales^(11,12,13)^.

Se argumenta que el marco de referencia de la complejidad puede sustentar un análisis sensible, crítico e integrador de la atención interprofesional a la salud de las mujeres indígenas en el contexto intercultural. Así, se plantea la pregunta: ¿cómo puede la teoría de la complejidad contribuir a la reflexión y a la práctica de la atención interprofesional a la salud de las mujeres indígenas en el ciclo grávido-puerperal?

De este modo, este estudio teórico-reflexivo, al dialogar con Edgar Morin, busca reflexionar, a la luz de la teoría de la complejidad, sobre las dinámicas de la atención interprofesional a la salud de las mujeres indígenas en el ciclo grávido-puerperal.

## MÉTODO

Se trata de un estudio teórico-reflexivo, de enfoque cualitativo, construido a partir del marco del Pensamiento Complejo de Edgar Morin, especialmente mediante el tetragrama de Morin (orden, desorden, interacción y organización) como marco analítico para revelar los encuentros, tensiones y posibilidades presentes en la atención interprofesional en contextos interculturales, en el marco de la asignatura «Construcción del Pensamiento Sistémico», cursada en el ámbito de la formación doctoral. Concebido inicialmente como capítulo de libro, el texto fue reelaborado y adaptado al formato de artículo científico-reflexivo.

El recorrido se basó en la articulación entre el marco teórico central y un corpus de producciones científicas nacionales e internacionales relacionadas con la atención interprofesional y la salud indígena. La selección de estas obras se realizó de manera intencional y analítica, considerando su preocupación temática, relevancia conceptual y potencial de diálogo con los supuestos del pensamiento/teoría de la complejidad/complejo.

Las búsquedas bibliográficas se realizaron en la Biblioteca Virtual en Salud (BVS), contemplando artículos, documentos técnicos y producciones teóricas pertinentes al campo de la salud colectiva, la enfermería y la salud indígena. No se trató de una revisión sistemática, sino de un estudio teórico orientado por la problematización reflexiva, con el objetivo de respaldar la construcción argumentativa y el análisis crítico propuesto.

La elaboración del estudio se llevó a cabo entre agosto y octubre de 2025, considerando las publicaciones disponibles hasta ese momento, lo que contribuye a la contextualización y actualidad de la reflexión desarrollada. El proceso analítico consistió en la lectura profunda, la interpretación crítica y la articulación conceptual de las obras seleccionadas, en diálogo continuo con el marco de referencia de Morin, con el objetivo de comprender la complejidad de la atención en salud en contextos marcados por la interprofesionalidad y la diversidad sociocultural.

Así, el presente estudio se perfila a partir del marco teórico-reflexivo de la teoría de la complejidad de Edgar Morin, con el fin de fundamentar la atención interprofesional de la salud de las mujeres indígenas en el ciclo grávido-puerperal.

### El Pensamiento Complejo de Edgar Morin

Como ya se ha presentado, el marco adoptado se basa en la teoría de la complejidad propuesta por Edgar Morin, filósofo, sociólogo y epistemólogo francés, nacido en 1921. Su biografía, marcada por pérdidas tempranas, como la muerte de su madre a los nueve años, contribuyó a una sensibilidad particular ante la condición humana y sus múltiples dimensiones. A lo largo de su trayectoria intelectual, Morin demostró interés por la literatura, la música y el cine, transitando por diferentes campos del conocimiento y consolidando una visión integradora, sobre todo en las ciencias humanas^(14)^.

La complejidad es un conjunto de elementos inseparablemente asociados, que se expresa como un tejido de acontecimientos, acciones, interacciones, retroacciones, determinaciones y casualidades que constituyen la realidad^(15)^. En este entrelazamiento están presentes incertidumbres, indeterminaciones y aleatoriedades que desafían las visiones reduccionistas o lineales^(16)^.

La teoría de la complejidad busca integrar aspectos que a menudo se consideran contradictorios: unidad y diversidad, continuidad y ruptura, construcción y deconstrucción, reconociendo que no es posible analizar las partes sin el todo, ni el todo sin las partes. En este sentido, la complejidad debe entenderse como una palabra-problema, y no como una palabra-solución: no proporciona respuestas listas, sino que invita a la reflexión permanente, al análisis crítico y a la apertura a lo inesperado^(15)^.

A la luz de la teoría de la complejidad, se refleja la coexistencia entre orden y desorden, ilustrando su idea con la observación del cielo estrellado. Lo que inicialmente parece una agrupación aleatoria de astros revela, con una mirada atenta, un orden cósmico; al mismo tiempo, al considerar el universo en expansión, se percibe el desorden creativo de estrellas que nacen, explotan y mueren. Así, el orden y el desorden constituyen dimensiones inseparables de lo real^(15)^.

Esta concepción es fundamental para abordar los desafíos de la atención de salud indígena en el ciclo grávido-puerperal, marcado por un complejo entramado de elementos culturales, espirituales, sociales y biológicos. La atención a las mujeres indígenas no puede reducirse a la lógica biomédica lineal, basada en protocolos técnicos; es necesario reconocer la coexistencia de múltiples órdenes y desórdenes, saberes tradicionales y científicos, rutinas institucionales y prácticas ancestrales, derechos conquistados y violencias históricas aún presentes^(1,17)^.

La teoría de la complejidad se sustenta en principios que se articulan en el circuito tetralógico o tetragrama —orden, desorden, interacción y organización—, orientando la reflexión hacia la reconexión de los saberes^(15)^. Los principios de la teoría de la complejidad, propuestos por Morin, ofrecen bases esenciales para comprender los fenómenos en su totalidad. El principio sistémico muestra que las partes y el todo son indisociables; el holográfico revela que cada parte contiene el todo y viceversa, superando reduccionismos y holismos. El circuito retroactivo y el recursivo destacan la autorregulación y la circularidad entre causas y efectos. La autonomía está siempre en relación de dependencia con el medio, mientras que el principio dialógico integra nociones opuestas que coexisten en la realidad. Por último, la reintroducción del conocimiento propone una reforma del pensamiento, reconociendo los límites, las incertidumbres y la necesidad de una contextualización constante.

En el ámbito de la salud indígena, los principios de la teoría de la complejidad de Morin ayudan a comprender el cuidado de manera ampliada. El principio dialógico pone de manifiesto la necesidad de integrar los conocimientos técnicos y los conocimientos tradicionales, permitiendo que la ciencia y la ancestralidad coexistan en diálogo. El principio recursivo muestra que las prácticas de cuidado no se agotan en sí mismas, sino que retroalimentan la cultura, la salud y el territorio, fortaleciendo continuamente la vida comunitaria. Por su parte, el principio hologramático revela que cada parte, ya sea el profesional, la comunidad, la mujer o la familia, contiene y refleja el todo, mostrando que el cuidado es simultáneamente individual y colectivo, singular y comunitario^(16,18)^.

### El Tetragrama de Morin y el Ciclo Grávido-Puerperal Indígena

A la luz de la teoría de la complejidad, los procesos dinámicos de la realidad pueden entenderse a través del tetragrama, una proposición que considera que cualquier sistema vivo o social es el resultado de la intersección entre orden, desorden, interacción y organización (Figura 1)^(15)^. Estos elementos no actúan de forma aislada, sino en constante movimiento de transformación. Relacionar el tetragrama con el ciclo grávido-puerperal de las mujeres indígenas permite reconocer la complejidad de este proceso en contextos interculturales y la necesidad de prácticas de salud ampliadas e inclusivas.

**Figura 1:** Tetragrama de Morin.

Fuente: Morin^(15:204)^.

A continuación, se propone una aproximación entre los elementos del tetragrama de la complejidad y la atención interprofesional en el ciclo de gestación y puerperio de las mujeres indígenas, reconociendo los límites de este análisis y su naturaleza parcial, abierta y en construcción, tal como exige el propio pensamiento complejo.

### Orden

El orden se refiere a los patrones establecidos, las normas culturales y los rituales que orientan el comportamiento social. Entre los pueblos indígenas, la gestación, el parto y el puerperio se llevan a cabo a partir de conocimientos tradicionales, costumbres ancestrales, funciones colectivas bien definidas y rituales estructurados de acuerdo con cada etnia. Esta orden promueve la cohesión comunitaria, el sentido de pertenencia y la seguridad simbólica para la gestante, quien se reconoce como parte de un ciclo mayor^(19,20)^.

En cambio, en el modelo biomédico, el orden está representado por protocolos estandarizados, flujos asistenciales y normativas técnicas, a menudo desconectados de las subjetividades y realidades socioculturales de las mujeres indígenas. Mientras que la lógica biomédica valora la regularidad y la previsibilidad, los saberes indígenas conciben la salud y la enfermedad como experiencias relacionales, que involucran al cuerpo, el territorio, la colectividad, los ancestros y las fuerzas espirituales^(21)^.

En el contexto del elemento «orden» del Tetragrama, la Política Nacional de Atención a la Salud de los Pueblos Indígenas (PNASPI) reconoce, aunque de manera limitada, la necesidad de articular las prácticas tradicionales y los conocimientos biomédicos, proponiendo un modelo de atención diferenciada e intercultural. Sin embargo, su implementación enfrenta innumerables desafíos, como la persistencia del etnocentrismo institucional y la fragmentación de las prácticas de atención^(22)^. Ejecutada por la Secretaría Especial de Salud Indígena (SESAI), esta política establece un modelo de atención diferenciada, con el objetivo de promover, proteger y recuperar la salud, garantizando el acceso al SUS y la articulación de servicios cercanos a las comunidades^(23,24)^.

La eficacia de la PNASPI exige tener en cuenta las especificidades culturales, geográficas y políticas de cada pueblo, así como la incorporación de tecnologías apropiadas. Los Distritos Sanitarios Especiales Indígenas (DSEI), actualmente con 34 unidades, son el eje estructurante de esta atención, integrándose al SUS a través de equipos multiprofesionales que incluyen a profesionales de la salud, educadores y antropólogos. En este marco, destacan los agentes indígenas de salud y los enfermeros, que actúan directamente en las aldeas, coordinando la atención con equipos itinerantes^(22,23)^.

La red de atención incluye centros base, servicios del SUS y Casas de Salud Indígena (CASAI), organizándose para garantizar una atención integral en todos los niveles de complejidad. Este modelo valora las prácticas culturales, la presencia de familiares, intérpretes y terapeutas indígenas, como las parteras, consolidándose como un espacio de atención intercultural e interprofesional^(22,23)^. Estudios recientes demuestran que, a pesar de la existencia de políticas amplias y constructivas, las acciones concretas se ven limitadas por diversos tipos de barreras, entre ellas las estructurales, logísticas y de comunicación^(11)^.

Las experiencias de las mujeres indígenas en el ciclo grávido-puerperal presentan singularidades arraigadas en valores culturales, ambientales y espirituales, que por su profundidad trascienden el conocimiento científico y cuestionan la linealidad de las prácticas convencionales^(1)^. A la luz de la complejidad, tales vivencias evidencian la necesidad de reconocer la interdependencia entre saberes distintos, integrando dimensiones biológicas, sociales, culturales y espirituales en una perspectiva plural^(25)^.

La idea de orden exige el diálogo con la idea de desorden; la idea enriquecida de orden recurre a las ideas de interacción y organización, sin excluir el desorden^(15)^.

### Desorden

El desorden representa tensiones, conflictos y rupturas. El encuentro entre la medicina occidental y los saberes indígenas genera con frecuencia choques de referencia, provocando incomodidades y tensiones interculturales. El traslado obligatorio de las gestantes a centros urbanos distantes ilustra este desorden, ya que rompe los vínculos con los rituales de cuidado y genera sufrimiento emocional. Sin embargo, según la teoría de la complejidad, el desorden también es potencia creativa, abriendo posibilidades de reorganización más inclusivas y equitativas^(15,25)^.

En el contexto de la atención a las mujeres indígenas, el desorden también se manifiesta en la incomprensión de los conocimientos tradicionales por parte de los profesionales, en la violación de los derechos reproductivos y en la sumisión a rutinas hospitalarias estandarizadas que ignoran los deseos y las prácticas culturales de las gestantes. Estas situaciones generan inseguridad, sufrimiento, desconexión simbólica y, a menudo, falta de atención^(11)^.

Es necesario aprender a pensar el orden y el desorden conjuntamente, entendiendo que el orden es relativo y relacional, mientras que el desorden está marcado por la incertidumbre^(15)^. Las políticas públicas de salud dirigidas a las poblaciones indígenas pueden estar relacionadas con una necesidad que el desorden ha despertado, siendo necesario pensar en entornos más acogedores y menos disruptivos para estas comunidades cuando necesitan atención fuera de las aldeas. Por lo tanto, el desorden, a la luz de la complejidad, no es sinónimo de caos destructivo, sino que representa la ruptura de patrones, el surgimiento de lo nuevo y lo imprevisible que desestabiliza estructuras consolidadas. El desorden, al perturbar el orden existente, crea oportunidades para la emergencia de otras configuraciones más complejas^(15)^.

Por lo tanto, el desorden también puede ser productivo. Cuando los profesionales de la salud se enfrentan a situaciones imprevistas, por ejemplo, rituales desconocidos, prácticas comunitarias no previstas en los protocolos o decisiones familiares que escapan a la lógica institucional, existe la posibilidad de escuchar y aprender. Esta apertura a lo inesperado puede poner en tensión las prácticas fragmentadas y generar espacios de atención más respetuosos y receptivos^(15)^.

En el ámbito interprofesional, el desorden se expresa en las tensiones entre los saberes disciplinarios, en los conflictos entre modelos de atención y en los límites comunicacionales de los equipos. Estas inestabilidades, cuando no se niegan, pueden fomentar diálogos transformadores, impulsando la co-construcción de estrategias más plurales y contextualizadas.

### Interacción

La interacción constituye el núcleo del tetragrama, articulando elementos distintos, en este caso, prácticas tradicionales y biomédicas. Esta articulación puede adoptar formas conflictivas o cooperativas, dependiendo de la disposición al diálogo, la escucha activa y el reconocimiento de saberes plurales. En el ciclo grávido-puerperal, las experiencias de partos humanizados en aldeas, en las que participan parteras, agentes indígenas de salud y equipos multiprofesionales, demuestran que la interacción puede transformar el desorden en oportunidad, reorganizando la atención interprofesional de manera culturalmente respetuosa^(26,27)^.

La interacción también se observa en el encuentro entre las gestantes indígenas y las instituciones de salud. Cuando este encuentro está marcado por la rigidez institucional y la negación de las especificidades culturales, las interacciones se convierten en fuente de conflicto y exclusión. Sin embargo, cuando existe mediación cultural, articulación con los líderes indígenas y flexibilidad institucional, se abren posibilidades para una atención más humana, negociada y compartida. Así, las políticas públicas culturalmente sensibles se vuelven imprescindibles, garantizando la interculturalidad y la participación activa de los pueblos indígenas en todas las etapas de planificación y ejecución, trabajando en conjunto con las diferencias^(13,18)^.

En el ámbito de la formación profesional, la interacción entre diferentes áreas del saber puede contribuir al fortalecimiento de las prácticas de cuidado, siempre que se reconozcan las asimetrías de poder, los prejuicios institucionales y la necesidad de construir un lenguaje común, sin dejar de lado las singularidades. El cuidado interprofesional va más allá de una técnica de organización del trabajo; se trata de una forma integral y ecológica de pensar y actuar, capaz de integrar dimensiones del conocimiento y de la práctica en un movimiento de práctica transformadora, con implicaciones conceptuales, metodológicas y políticas directamente relacionadas con el desarrollo del campo de la salud y la educación^(5)^.

La interprofesionalidad, caracterizada por el aprendizaje mutuo y el trabajo en equipo integrado, es crucial para superar la fragmentación de la atención y promover una atención de salud más integral y eficaz, alineada con los principios del Sistema Único de Salud (SUS)^(4)^. Estudios en Brasil y en la región indican que las adaptaciones culturales de la atención prenatal se asocian con un mejor proceso de atención y refuerzan estrategias para acelerar la reducción de la mortalidad materna basadas en la atención primaria de salud y el respeto cultural^(28)^.

En el ámbito de la salud, el trabajo interprofesional se revela como una vía para superar la fragmentación típica del modelo biomédico hegemónico, favoreciendo prácticas colaborativas, seguras e integradas. Esta perspectiva fortalece los vínculos entre equipos y comunidades, amplía la reciprocidad entre profesionales y permite acciones planificadas, contextualizadas y evaluadas colectivamente, orientadas a la atención integral.

Sin embargo, persiste el desafío de enfrentar las desigualdades y jerarquías históricas que aún estructuran los campos de la salud y la educación bajo lógicas colonizadas^(4,5)^.

En este escenario, la interprofesionalidad y la formación interdisciplinaria pueden asumir un carácter intercultural y emancipador, sobre todo cuando se aplican a la salud indígena en el ciclo grávido-puerperal. Inspirado en la teoría de la complejidad, este enfoque reconoce la inseparabilidad entre orden y desorden, técnica y tradición, ciencia y ancestralidad, abriendo espacio para prácticas de cuidado más sensibles, dialógicas y transformadoras^(5,10)^.

### Organización

De la dinámica entre orden, desorden e interacción surge la organización, que se traduce en la construcción de nuevos arreglos de atención, más inclusivos y adaptados a la realidad de las comunidades. Esta reorganización no es lineal, sino fruto de negociaciones constantes, incorporando elementos de la interculturalidad y la interprofesionalidad como estrategias para fortalecer la atención integral^(13,16)^. En el ciclo grávido-puerperal, esta organización se expresa en modelos de atención que reconocen a la mujer indígena como protagonista, articulando conocimientos técnicos y tradicionales para garantizar prácticas culturalmente seguras y socialmente justas.

La (re)organización, como cuarto elemento del tetragrama, es el resultado provisional de las interacciones entre orden, desorden e interacción. En lugar de representar una estabilidad absoluta, la organización en la teoría de la complejidad es transitoria, adaptativa y está en constante reconstrucción^(16)^. En la atención de la salud de las mujeres indígenas, las formas de organización del trabajo en salud deben reflejar esta dinámica. Los equipos interprofesionales deben actuar de manera flexible, colaborativa y sensible a las singularidades locales, superando los modelos jerárquicos y tecnocráticos que aún predominan en muchos servicios, así como la necesidad de prácticas comprometidas con el uso sostenible de los recursos naturales y con la comprensión de las interrelaciones entre los factores sociales, ambientales y de salud^(13,29)^.

Por lo tanto, una (re)organización de la atención basada en la complejidad implica reconocer a la mujer indígena como sujeto activo del proceso, integrando sus elecciones, conocimientos y experiencias en el ciclo grávido-puerperal. Implica, además, el fortalecimiento de redes de apoyo comunitario, el diálogo con parteras y líderes tradicionales, y la valorización del territorio como espacio generador de salud. La construcción de una organización relacional de la atención exige, además, la articulación entre políticas públicas, estrategias interprofesionales y dispositivos culturales específicos de cada contexto^(30)^. Se trata de un proceso inacabado, que requiere disposición para lidiar con incertidumbres, aprender de los errores y reorganizar continuamente las prácticas, como demuestra la Figura 2.

**Figura 2:** Representación del Tetragrama de Morin integrado al ciclo grávido-puerperal de las mujeres indígenas.

Fuente: autores, 2025.

## CONSIDERACIONES FINALES

A la luz de la teoría de la complejidad, este estudio permitió comprender que las dinámicas de la atención interprofesional a la salud de las mujeres indígenas en el ciclo grávido-puerperal se configuran como procesos relacionales, situados y no lineales, marcados por dimensiones culturales, espirituales, sociales y biológicas. La reflexión puso de manifiesto que esta atención se construye en la interacción entre diferentes saberes, prácticas profesionales y arreglos comunitarios, lo que exige enfoques que superen los modelos fragmentados y prescriptivos. Integrar la teoría de la complejidad a la salud indígena en el ciclo grávidopuerperal ofrece una lente crítica e integradora para comprender las múltiples dimensiones que atraviesan la salud materno-infantil. Reconocer la interconexión entre factores culturales, espirituales, sociales y biológicos permite construir prácticas de salud que respeten e integren los saberes tradicionales, promoviendo una atención más equitativa y eficaz. La articulación entre orden, desorden, interacción y organización permite comprender esta atención como un fenómeno dinámico, situado y profundamente relacional, que exige apertura a la pluralidad, sensibilidad ética y disposición para reinventar prácticas ante la complejidad cotidiana.

Desde el punto de vista de la práctica profesional, se sugiere la importancia de fortalecer estrategias de atención interprofesional basadas en la escucha de habilidades, en el reconocimiento de las autoridades comunitarias y en la articulación entre diferentes sistemas de conocimiento. Para la formación en salud, se destaca la necesidad de invertir en procesos formativos interculturales e interprofesionales, que incorporen el pensamiento complejo como herramienta pedagógica, favoreciendo la formación de profesionales sensibles a las dimensiones éticas, políticas y culturales del cuidado en contextos indígenas. En el ámbito de las políticas públicas, el estudio refuerza la urgencia de planificar e implementar acciones que superen los modelos biomédicos centrados exclusivamente en protocolos e indicadores, avanzando en la construcción de políticas culturalmente seguras, territorializadas y acordadas con las comunidades. Superar los desafíos requiere no solo políticas públicas adecuadas, sino también formación intercultural para los profesionales de la salud, a fin de garantizar que las mujeres indígenas tengan acceso a una atención que respete su identidad, promueva el bienestar y fortalezca los vínculos comunitarios.

Se reconocen, sin embargo, las limitaciones epistemológicas inherentes al método teórico-reflexivo adoptado. La ausencia de una búsqueda sistemática en la literatura y la inexistencia de un diálogo empírico directo con experiencias concretas de atención pueden restringir el alcance de las reflexiones, que se sostienen predominantemente en el plano conceptual e interpretativo. La inmensa diversidad cultural y social de los pueblos indígenas en Brasil constituye una limitación para las reflexiones presentadas en este estudio. Las tensiones y posibilidades del cuidado interprofesional varían ampliamente entre diferentes etnias y regiones, y esta reflexión no puede generalizarse a la totalidad de los contextos interculturales. Las contribuciones de este estudio al avance de la ciencia de la enfermería están relacionadas con la propuesta de un pensamiento complejo que permite desvelar los encuentros, tensiones y posibilidades presentes en el cuidado interprofesional de las mujeres indígenas.

## DISPONIBILIDAD DE DATOS

Todo el conjunto de datos que respalda los resultados de este estudio se publicó en el propio artículo.
